# Do We Still Have a Digital Divide in Mental Health? A Five-Year Survey Follow-up

**DOI:** 10.2196/jmir.6511

**Published:** 2016-11-22

**Authors:** Dan Robotham, Safarina Satkunanathan, Lisa Doughty, Til Wykes

**Affiliations:** ^1^ Department of Psychology Institute of Psychiatry, Psychology & Neuroscience King's College London London United Kingdom

**Keywords:** digital divide, socioeconomic factors, technology, mobile phone, psychotic disorders, distance counseling

## Abstract

**Background:**

Nearly everyone in society uses the Internet in one form or another. The Internet is heralded as an efficient way of providing mental health treatments and services. However, some people are still excluded from using Internet-enabled technology through lack of resources, skills, and confidence.

**Objective:**

Five years ago, we showed that people with severe mental illness were at risk of digital exclusion, especially middle-aged patients with psychosis and/or people from black or minority ethnic groups with psychosis. An understanding of the breadth of potential digital exclusion is vital for the implementation of digital health services. The aim of this study is to understand the context of digital exclusion for people who experience mental illness.

**Methods:**

We conducted a survey involving people with a primary diagnosis of psychosis or depression in London, United Kingdom. A total of 241 participants were recruited: 121 with psychosis and 120 with depression. The majority of surveys were collected face-to-face (psychosis: n=109; depression: n=71). Participants answered questions regarding familiarity, access, use, motivation, and confidence with Internet-enabled technologies (ie, computers and mobile phones). Variables predicting digital exclusion were identified in regression analyses. The results were compared with the survey conducted in 2011.

**Results:**

Digital exclusion has declined since 2011. Online survey collection introduced biases into the sample, masking those who were likely to be excluded. Only 18.3% (20/109) of people with psychosis in our sample were digitally excluded, compared with 30% (28/93) in 2011 (χ^2^_1_=3.8, *P*=.04). People with psychosis had less confidence in using the Internet than people with depression (χ^2^_1_=7.4, *P*=.004). Only 9.9% (24/241) of participants in the total sample were digitally excluded, but the majority of these people had psychosis (n=20). Those with psychosis who were digitally excluded were significantly older than their included peers (*t*_30_=3.3, *P*=.002) and had used services for longer (*t*_97_=2.5, *P*=.02). Younger people were more likely to use mobile phones. Digitally excluded participants cited a lack of knowledge as a barrier to digital inclusion, and most wanted to use the Internet via computers (rather than mobile phones).

**Conclusions:**

Digital exclusion is lower, but some remain excluded. Facilitating inclusion among this population means helping them develop skills and confidence in using technology, and providing them with access. Providing mobile phones without basic information technology training may be counterproductive because excluded people may be excluded from mobile technology too. An evidence-based digital inclusion strategy is needed within the National Health Service to help digitally excluded populations access Internet-enabled services.

## Introduction

Online services are integral to the future of the UK National Health Service (NHS) [1]. The Internet is almost ubiquitous. Between 2000 and 2016, worldwide use increased by 900% [2]; 86% of the UK population have access and more than three-quarters use it on a near-daily basis [3]. Nonetheless, these figures hide a digitally excluded minority: approximately 10% of the UK population have never used the Internet [4]. Reducing digital exclusion in this minority has been highlighted as an NHS priority [5].

Mental illness, particularly depression and anxiety, have long been targets for online interventions. Cognitive behavioral therapy tools have been available online for many years, such as “Beating the Blues.” Computer literacy and familiarity with the Internet are essential for online interventions to be effective. A survey conducted in 2011 of people with mental illness (the majority of whom experienced psychosis) demonstrated that people who had been unwell for longer were at risk of digital exclusion, and that service users from black and minority ethnic (BME) groups were more likely to access public Internet facilities rather than personal devices [6], which may affect the privacy of their health data. Other studies indicate that people with longer-term psychotic illnesses have showed higher rates of independent use of digital tools than people using early intervention services [7]

The nature of the digital divide is complex and varies over time [8]. Technological developments in the last five years have been dramatic; more than three-quarters of the UK population now own an Internet-enabled mobile phone [9], whereas traditional public sources of the Internet (eg, libraries) are suffering from reduced funding [10]. There is a lack of recent information on digital exclusion in those who use mental health services, and this information is important for those developing and implementing eHealth services and therapies [11-13]. A recent online survey of people with psychosis identified high proportions accessing Internet-enabled devices, but it only included people who were already using the Internet [14]. The aim of this study is to update conceptions of digital exclusion in people with two different mental illness diagnoses (psychosis and unipolar depression). The hypotheses of the study were

1. Digital exclusion is less in 2016 than in 2011 due to the increased availability of Internet-enabled mobile phones;

2. People with psychosis are at higher risk of exclusion compared to people with depression; and

3. People at higher risk of digital exclusion (eg, people with psychosis, those with a longer duration of illness, people from BME groups) will still show higher rates of exclusion.

In addition, we wanted to understand the sorts of barriers that need to be overcome to make any digital health service available to the largest proportion of patients.

## Methods

### Design and Setting

We collected data from a cross-sectional survey of Internet technology use among people with a primary diagnosis of psychosis or unipolar depression. The study took place at a large UK secondary mental health care provider. Data were compared with the same data collected in a 2011 survey [6].

### Sample and Recruitment

We recruited participants with a primary clinical diagnosis of either psychosis (schizophrenia or schizoaffective disorder) or unipolar depression, confirmed through case notes.

### Measures

We collected demographic and clinical data via survey and from case notes.

The Digital Inclusion Survey included items from the 2011 version [6]. The domains of Internet use included were Internet access, familiarity, confidence, daily use, and motivation to use Internet-enabled technology. The Digital Inclusion Survey included items on barriers to using technology, including lack of knowledge, availability, lack of credit on pay-as-you-go phones (or lack of money to purchase credit), Internet access, wanting to use technology, and security concerns. We also investigated the use of social media.

Survey terminology was updated or adapted to reflect technology developments since 2011. The survey could be completed face-to-face or online. It was assessed for acceptability and feasibility with service users who were attending “drop-in” Internet practice sessions organized by the NHS Trust [15].

### Procedure

To access those who have different patterns of service use, we recruited participants from inpatient units, outpatient community psychosis teams, early intervention services (for psychosis), and community services for people with depression. We also used an online research register [16]. All register participants were contacted using their preferred method of contact; those who responded by email were offered the option of completing the survey online.

Ethical approval was granted by the London Camden and Kings Cross Research Ethics Committee (reference: 10/H0722/79).

### Data Analysis

#### Sampling Effects

We explored whether the diagnosis samples differed in their characteristics and across different methods of data collection (face-to-face or online).

#### Exploring Internet Use

Chi-square tests (two-sided) were used to explore differences in Internet use and motivation to use the Internet between diagnostic groups. Descriptive statistics were used to analyze self-reported barriers to Internet access and use of social media. Those who completed the survey face-to-face were analyzed separately from those who completed it online.

#### Characterizing Digital Exclusion

“Lacking access to Internet technology” and “lacking confidence in using Internet technology” are potential indicators of digital exclusion. Logistic regression analyses were conducted to identify whether three candidate variables (each identified from previous research) predicted exclusion: age, ethnicity, and chronicity of illness. We completed separate regression analyses to see if these factors predicted exclusion from (1) all Internet-enabled devices, (2) computers, and (3) Internet-enabled mobile phones.

Digital exclusion was defined as anyone lacking access to any Internet-enabled device (or lacking confidence in using any Internet-enabled device) *and* accessing social media sites infrequently (ie, monthly or less than monthly). We investigated the characteristics of this group and examined differences between this group and the remainder using chi-square tests.

#### Examining Differences Over Time

People with psychosis were compared with those from the 2011 sample. To ensure consistency across the samples, we only included participants from the 2011 survey who had a primary clinical diagnosis of schizophrenia or schizoaffective disorder, and excluded participants from the 2016 sample who had completed the survey online. Two-sided *t* tests and chi-square tests were used to compare demographics. One-sided chi-square tests were used to compare digital exclusion over time.

## Results

### Sample Characteristics

A total of 241 participants were recruited: 166 through visits to outpatient clinical teams, 22 from inpatient units, and 53 from research registers. Demographic and clinical information (along with comparisons between the two diagnostic groups) are presented in Table 1.

**Table 1:** Sample characteristics.

Variable		2011	2016^a^				
		Psychosis (n=93)	Psychosis (n=121)	Depression (n=120)	χ^2^_1_	*t* (df)	*P* (2-sided)
Age (years), mean (SD)		34.6 (11.6)	38.2 (13.2)	39.1 (13.4)		0.55 (234)	.59
Gender (male), n (%)		65 (70)	81 (67)	53 (44)	12.7		<.001
Illness duration (years), mean (SD)		—	10.1 (10.0)	4.3 (6.9)		–4.97 (215)	<.001
**Ethnicity, n (%)**							
	BME	58 (62)	75 (62)	29 (24.2)			
	White	35 (38)	46 (38)	88 (73.3)			
**Location, n (%)**							
	Community team	67 (72)	67 (55.4)	24 (20)			
	Early intervention	24 (26)	27 (22.3)	73 (60.8)			
	Other	—	27 (22.3)	23 (19.2)			

^a^ Comparisons made within 2016 sample only.

The groups were balanced for age, but those with psychosis had a longer history of illness. The psychosis group contained more people from BME backgrounds (χ^2^_1_=33.5, *P<*.001) and more men.

In all, 180 participants completed the survey face-to-face (109 people with psychosis, 71 with depression) and 61 completed the survey online (12 with psychosis, 49 with depression). There were no significant demographic differences between different modes of completion for either diagnostic group. Among people with psychosis only, those who completed the online survey had more confidence with computers (χ^2^_1_=3.7, *P*=.07), better access to mobile phones (χ^2^_1_=4.5, *P*=.06), and were more confident using a mobile phone (χ^2^_1_=3.6, *P*=.07). These tests showed imbalances within the sample despite the fact that these associations did not reach statistical significance. Therefore, subsequent analysis of Internet use was completed separately for the two modes of survey completion.

### Exploring Internet Use

**Figure 1:**
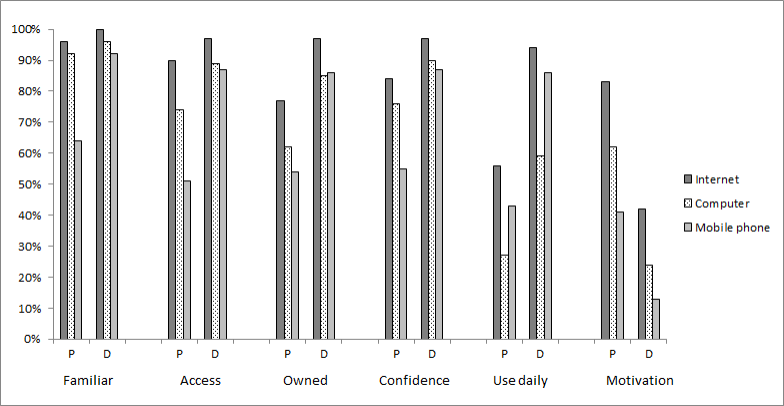
Proportion of people with a diagnosis of psychosis (P; n=109) or depression (D; n=71) using Internet-enabled devices in 2016.

For face-to-face survey completion, Internet use among people with psychosis and depression is presented in Figure 1. Fewer people with psychosis had access to the Internet (χ^2^_1_=3.4, *P*=.08), either via computers (χ^2^_1_=5.6, *P*=.02) or mobile phones (χ^2^_1_=24.6, *P*<.001). Fewer people with psychosis were confident in using the Internet (χ^2^_1_=7.4, *P*=.004) with computers (χ^2^_1_=5.6, *P*=.02) or mobile phones (χ^2^_1_=20.5, *P*<.001). Conversely, people with psychosis had higher motivation to increase their use of the Internet (χ^2^_1_=31.5, *P*<.001), computers (χ^2^_1_=25.5, *P*<.001), and mobile phones (χ^2^_1_=16.8, *P*<.001) than those with depression. There was a significant negative correlation between Internet access and desire to increase Internet use (*r*=–.152, *P=*.04). This suggests that those who used the Internet already did not want to increase their use (or they were already using it frequently). Only one association was significant when looking at the participants who completed the survey online: people with psychosis had higher motivation to increase their use of the Internet than people with depression (χ^2^_1_=5.1, *P*=.03).

For face-to-face survey completion (psychosis: n=109), the most common barriers to using the Internet were security concerns (45.9%, 50/109), lack of credit/money (45%, 49/109), lack of knowledge (40.4%, 44/109), lack of places to access the Internet (35.8%, 39/109), and lack of availability (33.9%, 37/109). Only 15.6% (17/109) cited not wanting to use the Internet as a barrier. Among the equivalent sample with depression (n=71), the most common barriers to using the Internet were security concerns (49%, 35/71) followed by lack of credit/money (30%, 21/71). The same concerns were evident in the individuals who completed the online survey.

For people with psychosis, 55% (60/109) reported having a social media account (eg, Facebook or Twitter), 32.1% (35/109) used social media at least daily, and 45.9% (50/109) the sample reported never using it. In comparison, 82% (58/71) of the depression sample had a social media account, with 63% (45/71) using it at least daily and only 16% (11/71) never using it. The pattern of results in the online sample was similar for people with depression, but a higher proportion of people with psychosis in the online sample had a social media account (10/12) and used social media at least daily (9/12).

### Characterizing Digital Exclusion: Who Is Excluded?

Older age predicted reduced confidence with mobile phones for people with psychosis (beta=–.1, OR 0.90, 95% CI 0.84-0.95, *P*<.001), reduced access to mobile phones for people with psychosis (beta=–.05, OR 0.95, 95% CI 0.91-0.99, *P*=.04), and reduced access to computers for people with depression (beta=–.12, OR 0.89, 95% CI 0.80-0.98, *P*=.02). Ethnicity and duration of service use did not predict digital exclusion.

In all, 24 participants met the criteria for digital exclusion, of which 23 completed the survey face-to-face. Twenty digitally excluded participants had a primary diagnosis of psychosis. In comparison to “digitally included” peers, this group was significantly older (excluded: mean 45.7, SD 9.7 years; included: mean 36.8, SD 12.7 years; *t*_30_=3.3, *P*=.002; equal variances not assumed) and had used mental health services for longer (excluded: mean 14.1, SD 9.2 years; included: mean 8.7, SD 8.3 years; *t*_97_=2.5, *P*=.02). There were no other clinical or demographic differences. Table 2 shows that the rate of digital exclusion was higher in older people, in those with longer-term illnesses, and in people from BME groups. This was the case in both diagnostic groups.

**Table 2:** Digital exclusion according to key variables.

Group variable	Psychosis		Depression	
	n	Excluded, n (%)	n	Excluded, n (%)
Age (≥36 years)	62	15 (24)	37	3 (8)
Age (<36 years)	44	3 (7)	32	0
Duration of illness (≥3 years)	74	17 (23)	27	2 (7)
Duration of illness (<3 years)	25	1 (4)	37	0
White	41	6 (15)	48	1 (2)
BME	54	14 (21)	19	2 (10)

Despite being digitally excluded, people with psychosis said that they wanted to use the Internet more often (yes=17, no=3), particularly through computers (yes=16, no=4) rather than mobile phones (yes=8, no=12). The most commonly reported barrier to using the Internet was lack of knowledge followed by lack of credit/money.

Likewise, those with depression who met the criteria for digital exclusion (n=4), were older (excluded: mean 53, SD 11 years; included: mean 39, SD 13 years) and had been using mental health services for longer (10 years vs 4 years). The sample was too small to test for statistical significance. The pattern of motivation to increase use and to use Internet-enabled devices appeared similar to that of the psychosis group.

### Examining Digital Exclusion Over Time

The only significant difference between the 2011 and the 2016 samples was in age; the 2011 sample were younger (2011: mean 34.6, SD 11.6 years; 2016: n=106, mean 38.3, SD 12.7 years; *t*_197_=2.1, *P*=.03).

Only 18.3% (20/109) of people with psychosis in the 2016 sample were digitally excluded compared to 30% (28/93) from 2011 and this difference was statistically significant (χ^2^_1_=3.8, *P*=.04). The demographics of the digitally excluded group in the 2011 were similar to those of the excluded group in 2016, in terms of age, gender, and proportion from a BME background. No differences were found in access or confidence with computers. However, there was a significant increase in mobile phone access (χ^2^_1_=6.7, *P*=.01) and confidence in using them (χ^2^_1_=28.8, *P*<.001). People were more motivated to use technology in 2016 than in 2011 with 62.4% (68/109) wanting to increase use of computers in 2016 compared to 48% (45/93) in 2011 (χ^2^_1_=4.0, *P*=.03). Equivalent figures for mobile phones were 41.3% (45/109) and 18% (17/93), respectively (χ^2^_1_=12.5, *P*<.001).

The 2016 sample showed a greater proportion used the Internet daily (56%, 61/109) compared to 35% (33/93) in 2011 and this difference was significant (χ^2^_1_=8.5, *P=*.005). This is due to the increase in the daily use of Internet-enabled mobile phones to 43.1% (47/109), up from 9% (8/93) in 2011 (χ^2^_1_=30.2, *P*<.001), as there were no significant differences in daily computer use across the samples.

## Discussion

Two new findings appear since the last time we carried out this survey in 2011. First, only collecting data from online surveys is likely to produce biased results, particularly among people with psychosis. Second, digital exclusion has decreased over time, but has not disappeared. The methodological differences are important because new methods of providing mental health services using mobile devices depend on data estimating breadth of coverage. Studies only using online surveys [14] overestimate digital inclusion, access, and confidence with Internet-enabled devices among people with psychosis. The fact that there has been a reduction in digital exclusion is to be celebrated, but the fact that older individuals with more chronic conditions (eg, psychosis) have higher rates of digital exclusion is pause for thought. These people are the exact group who may benefit the most from digital health support to supplement current care.

The good news is that the majority of the 2016 sample claimed to have Internet access. The bad news is that digital inequality still exists. Daily use in the general UK population has been reported as 78% [3], higher than in the sample of people with psychosis. People with psychosis reported less confidence, access, use, and familiarity with the Internet (and devices) than people with depression. They also reported higher motivation to use the Internet more often.

There is still a digitally excluded minority without access to any Internet-enabled device and/or without confidence in using the Internet, and their characteristics were similar to those identified five years previously. They are excluded because they lack the knowledge, skill, and financial resources, not because they lack the willingness. This echoes previous findings [6,17,18], although the digitally excluded sample was even more motivated to use technology than five years ago.

The prevalence of Internet-enabled mobile phones has been the major change in online habits in the last five years, the potential of mobile phones for reducing digital exclusion cannot be taken for granted. Mobile phone use has been mainly adopted by younger service users who suffer less from digital exclusion and are more likely to use social media [19]. Individuals who are digitally excluded also preferred the idea of connecting to the Internet via a computer rather than a mobile phone. Mobile technology itself can exclude middle-aged and older people with psychosis [20], which explains our finding relating to the lack of motivation to use the Internet through mobile phones among digitally excluded individuals. If this is the case, then mobile phones could further exclude those who are already digitally excluded.

This study shows how difficult it is to overcome the digital divide. Providing digitally excluded people with mobile phones will not facilitate inclusion. For people who have never used the Internet, a mobile phone may initially seem even more daunting than a computer. Reducing digital exclusion will undoubtedly require training on mobile phones, but also requires intermediate steps. This may include training in basic Internet skills (possibly using computers), through the facilitation of structured information technology skills classes delivered specifically as part of a wider community service for people with psychosis [15]. This is possible to complete in conjunction with training in the use of mobile phones and the mobile Internet, as has been shown successfully in the past [13].

The sample is large enough to identify subgroups for analysis, but low numbers of digitally excluded people limit the power of statistical tests for the subsample. There were demographic differences between diagnostic groups, but such differences reflect demographic differences in the prevalence of these illnesses. Lastly, the 2011 sample was slightly (but significantly) younger than the 2016 sample, which adds further support to our findings because younger people are less likely to be digitally excluded.

Digital exclusion is common enough to cause problems for health service providers working with particular populations. It occurs more frequently among long-term users of psychosis services. The development of eHealth interventions in psychosis must account for this. Although the majority will be able to use these services, a minority will need extra support and the opportunity to learn basic information technology skills. An evidence-based digital inclusion strategy is needed to prevent digitally excluded populations becoming excluded from increasingly digital NHS services and from society in general.

## Abbreviations

BME: black and minority ethnic
NHS: National Health Service
